# Artificial intelligence for the detection of airway nodules in chest CT scans

**DOI:** 10.1007/s00330-025-11468-6

**Published:** 2025-03-05

**Authors:** Ward Hendrix, Nils Hendrix, Ernst T. Scholten, Bram van Ginneken, Mathias Prokop, Matthieu Rutten, Colin Jacobs

**Affiliations:** 1 Diagnostic Image Analysis Group, Department of Medical Imaging, Radboud University Medical Center, Nijmegen, The Netherlands; 2 Department of Radiology, Jeroen Bosch Hospital, ‘s-Hertogenbosch, The Netherlands; 3 Jheronimus Academy of Data Science, ‘s-Hertogenbosch, The Netherlands; 4 Department of Radiology, University Medical Center Groningen, Groningen, The Netherlands

**Keywords:** Thorax, Lung neoplasms, Tracheal neoplasms, Tomography (X-ray computed), Artificial intelligence

## Abstract

**Objectives:**

Incidental airway tumors are rare and can easily be overlooked on chest CT, especially at an early stage. Therefore, we developed and assessed a deep learning-based artificial intelligence (AI) system for detecting and localizing airway nodules.

**Materials and methods:**

At a single academic hospital, we retrospectively analyzed cancer diagnoses and radiology reports from patients who received a chest or chest–abdomen CT scan between 2004 and 2020 to find cases presenting as airway nodules. Primary cancers were verified through bronchoscopy with biopsy or cytologic testing. The malignancy status of other nodules was confirmed with bronchoscopy only or follow-up CT scans if such evidence was unavailable. An AI system was trained and evaluated with a ten-fold cross-validation procedure. The performance of the system was assessed with a free-response receiver operating characteristic curve.

**Results:**

We identified 160 patients with airway nodules (median age of 64 years [IQR: 54–70], 58 women) and added a random sample of 160 patients without airway nodules (median age of 60 years [IQR: 48–69], 80 women). The sensitivity of the AI system was 75.1% (95% CI: 67.6–81.6%) for detecting all nodules with an average number of false positives per scan of 0.25 in negative patients and 0.56 in positive patients. At the same operating point, the sensitivity was 79.0% (95% CI: 70.4–86.6%) for the subset of tumors. A subgroup analysis showed that the system detected the majority of subtle tumors.

**Conclusion:**

The AI system detects most airway nodules on chest CT with an acceptable false positive rate.

**Key Points:**

***Question*** *Incidental airway tumors are rare and are susceptible to being overlooked on chest CT*.

***Findings*** *An AI system can detect most benign and malignant airway nodules with an acceptable false positive rate, including nodules that have very subtle features*.

***Clinical relevance*** *An AI system shows potential for supporting radiologists in detecting airway tumors*.

## Introduction

Tracheobronchial or airway cancers are a rare form of cancer and constitute less than 5% of all cancers of the lower respiratory tract [1, 2]. They are usually diagnosed at an advanced stage, because early symptoms (e.g., cough, chest pain, and wheezing) are non-specific and can be misinterpreted as a lung infection, chronic obstructive pulmonary disease (COPD), or asthma [3, 4]. Another reason for the delay in diagnosis is that airway abnormalities are hard to detect on chest radiographs as they are often obscured by mediastinal structures [5, 6]. As a result, the patient’s prognosis is poor and the five-year survival is only 12–15% [1, 2]. The prognosis substantially improves when the cancers are diagnosed at an early stage and surgical resection is possible [1, 7]. Early diagnosis is also important for their benign counterparts, since these slow-growing, obstructing lesions can remain unrecognized for months or even years while patients undergo prolonged treatment for COPD or asthma [8, 9].

Computed tomography (CT) is the preferred imaging modality for examining the trachea and bronchi before further clinical evaluation with bronchoscopy [3, 5]. However, even if a CT scan is available, early-stage airway tumors are known to be blind spots for radiologists [10–13]. In a study by White et al, it was found that 67% of missed lung cancers (10/15) on routine clinical CT scans were located in the bronchi [11]. Remarkably, overlooked endobronchial tumors were not small (mean diameter of 1.2 cm) and were sometimes overlooked in patients with a highly suggestive clinical history (e.g., hemoptysis). Similar findings were reported by Scholten et al [12] who investigated missed lung cancers in a large lung cancer screening trial. They found that most detection errors occurred with endobronchial nodules, which accounted for 22% of the missed cancers (5/22) in the trial. It has been argued that human error is a major contributor to these detection failures: observers are mainly focused on the far more common nodules in the lung parenchyma and may get distracted by major findings elsewhere in the chest.

In the context of recent advancements in artificial intelligence (AI) [14], deep learning-based computer-aided detection (DL-CAD) systems could be promising to aid radiologists in detecting airway tumors. For the task of lung nodule detection, DL-CAD systems can achieve a high sensitivity of 88–92% with one false positive per scan on average in a routine clinical setting [15–17]. It has been demonstrated that radiologists can obtain a higher detection sensitivity with DL-CAD systems as concurrent or second readers [18, 19]. DL-CAD systems may be especially useful in airway tumor detection, as these systems are not subject to the cognitive biases discussed previously.

To the best of our knowledge, no DL-CAD system has been evaluated for the detection of airway nodules despite their high clinical relevance. These nodules are assigned a high-risk level in nodule management guidelines for lung cancer screening [20, 21], which were recently updated for airway nodules. In this study, we develop a DL-CAD system and evaluate its technical performance for the detection and localization of airway nodules in a routine clinical setting.

## Materials and methods

### Datasets

This retrospective study was approved by the local institutional review board of Radboud University Medical Center (Radboudumc) in the Netherlands. In consideration of the retrospective design and use of pseudonymized data, the requirement for informed consent was waived. A dataset of 320 chest and chest–abdomen CT scans, each obtained from a unique patient between 2004 and 2020, was collected from the picture archiving and communication system (PACS). These scans were utilized to train and validate a deep learning-based system for the detection of airway nodules. Cancer diagnoses from 2000 to 2020 were obtained from the National Netherlands Cancer Registry (NCR). The AI system was pre-trained on the publicly available LUNA16 dataset [22] for intrapulmonary nodule detection.

**Fig. 1:**
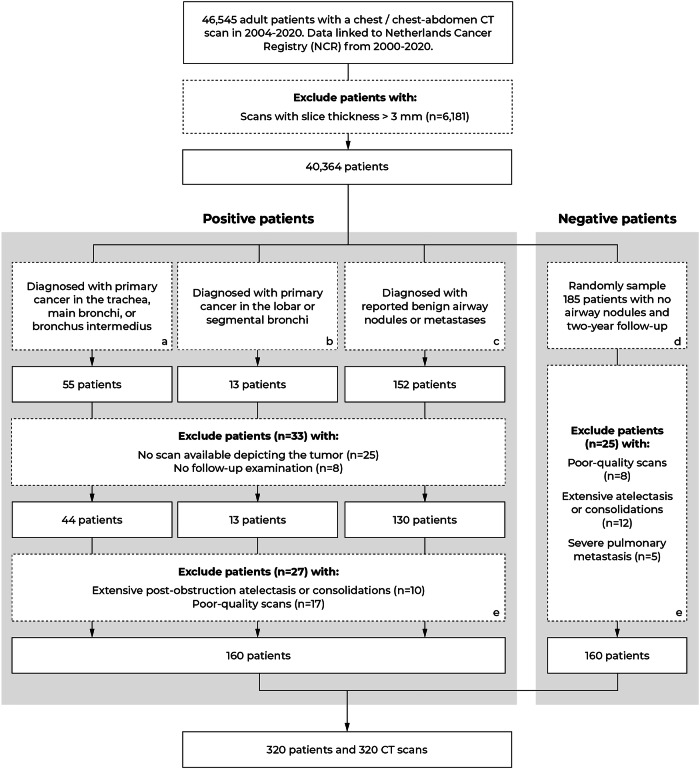
Data selection flowchart. ^a^Patients were pre-selected with ICD-O topology codes C339 and C340 before verification. ^b^Patients were pre-selected with ICD-O topology codes C341–C349 and a natural language processing (NLP) analysis before verification (see Appendix 2). ^c^Patients were pre-selected with an NLP analysis before verification (see Appendix 2). ^d^Negative patients were sampled from 2004 to 2018 due to two-year follow-ups. ^e^Poor-quality scans have severe breathing artifacts, missing slices, or partially depict airway nodules

A study selection flowchart for the airway nodule dataset is shown in Fig. 1. An experienced radiologist (E.T.S., 32 years of experience with chest CT) analyzed the scans according to the eligibility criteria as defined in the next section. Patients with tracheobronchial cancer were included based on the ICD-O [23] topography codes. Codes C339 and C340 were used to identify patients with cancers in the large airways (i.e., trachea, carina, main bronchus, bronchus intermedius). Codes C341–C349, encompassing intrapulmonary cancers, were used for pre-selecting patients with cancer in the smaller airways (i.e., lobar and (sub)segmental bronchi). Next, a natural language processing (NLP) analysis of radiology reports was applied to refine the selection by distinguishing airway cancers from intrapulmonary cancers. Patients with benign airway nodules (e.g., mucus secretion, benign neoplasms) or metastases were pre-selected with an NLP analysis of radiology reports and then verified with additional clinical information (e.g., biopsy and surgical information). We randomly sampled patients without airway nodules (with two-year follow-up) and matched the size of this group to the number of patients with airway nodules. For each patient, the earliest CT scan featuring an airway nodule, or the first available scan was chosen for analysis. Table 1 and Supplementary Table 1 (Appendix 1) show the dataset characteristics and additional imaging parameters, respectively. Table 2 provides an overview of the nodule characteristics. Appendix 2 (supplement) provides detailed information about the NLP analysis.

**Table 1:** Dataset characteristics

Characteristic	All	With airway nodules	Without airway nodules
Period	2004–2020	2004–2020	2004–2018
No. of patients	320	160	160
Sex—no. (%)			
Men	182 (56.9)	102 (63.8)	80 (50.0)
Women	138 (43.1)	58 (36.3)	80 (50.0)
Age—median (IQR)			
All	62 (51–70)	64 (54–70)	60 (48–69)
Men	62 (52–70)	65 (57–70)	60 (45–67)
Women	60 (48–70)	60 (46–66)	61 (49–73)
No. of CT scans	320	160	160
Protocol—no. (%)			
Chest	192 (60.0)	126 (78.8)	94 (58.8)
Chest–abdomen	128 (40.0)	34 (21.2)	66 (41.2)
Contrast-enhanced—no. (%)			
Yes	185 (57.8)	84 (52.5)	101 (63.1)
No	135 (42.2)	76 (47.5)	59 (36.9)
Slice thickness in mm—median (IQR)	1.0 (1.0–2.5)	1.0 (1.0–3.0)	1.0 (1.0–2.0)
Axial plane resolution in mm—median (IQR)	0.73 (0.67–0.78)	0.71 (0.67–0.78)	0.74 (0.68–0.78)

**Table 2:** Nodule characteristics

Characteristic	Categories				
	All airway nodules	Primary airway cancers	Airway metastases	Benign airway tumors	Non-tumorous airway nodules^a^
Patients—no. (%)^b^	160	65 (40.6)	10 (6.2)	13 (8.1)	78 (48.8)
Lesions—no. (%)	186	67 (36.0)	12 (6.5)	13 (7.0)	94 (50.5)
Nodules per scan—median (IQR)	1 (1–1)	1 (1–1)	1 (1–2)	1 (1–1)	1 (1–2)
Basis of diagnosis—no. (%) of nodules^c^					
Bronchoscopy with biopsy or cytologic testing	96 (51.6)	67 (100)	7 (58.3)	12 (92.3)	10 (10.6)
Bronchoscopy with neither biopsy nor cytologic testing	23 (12.4)		1 (8.3)		22 (23.4)
Imaging follow-up	67 (36.0)		4 (33.3)	1 (7.7)	62 (66.0)
Diameter in mm—median (IQR)^d^	9.2 (6.4–13.6)	14.5 (10.1–19.8)	10.9 (7.1–14.9)	10.5 (7.3–13.9)	7.1 (5.1–9.3)
Volume in mm^3^—median (IQR)^d^	410 (140–1313)	1580 (547–4084)	695 (190–1752)	612 (204–1396)	190 (69–416)
Density in HU—median (IQR)^d^	−5.5 (−62.6 to 47.3)	52.0 (13.0–76.5)	40.5 (20.3–58.5)	−27.0 (−70.0 to 26.0)	−38.3 (−94.8 to −15.3)
Site—no. (%) of nodules					
Trachea	81 (43.5)	20 (29.9)		2 (15.4)	59 (62.8)
Right main bronchus	33 (17.7)	15 (22.4)	3 (25.0)	2 (15.4)	13 (13.8)
Left main bronchus	27 (14.5)	8 (11.9)	3 (25.0)	4 (30.8)	12 (12.8)
Right upper lobe	7 (3.8)	4 (6.0)	1 (8.3)	1 (7.7)	1 (1.1)
Right middle lobe	2 (1.1)				2 (2.1)
Right lower lobe	13 (7.0)	7 (10.4)	2 (16.7)	2 (15.4)	2 (2.1)
Left upper lobe	11 (5.9)	6 (9.0)	2 (16.7)	1 (7.7)	2 (2.1)
Left lower lobe	12 (6.5)	7 (10.4)	1 (8.3)	1 (7.7)	3 (3.2)
Tumor score—no. (%) of nodules^e^					
Obvious		20 (29.9)	3 (25.0)	2 (15.4)	
Relatively obvious		21 (31.3)	2 (16.7)	5 (38.5)	
Subtle		10 (14.9)	4 (33.3)	2 (15.4)	
Very subtle		16 (23.9)	3 (25.0)	4 (30.8)	

^a^ Includes mostly mucus secretion (≥ 94%), but also blood, scarring, or amyloidosis

^b^ Note that six patients had multiple nodules from different categories

^c^ Includes both diagnostic and interventional bronchoscopy. If applicable, the basis of diagnosis for the metastasis site is provided. The primary site of all metastases was confirmed with either histopathological or cytopathological evidence

^d^ These characteristics are based on 3D nodule segmentations. Diameter denotes the equivalent diameter. Density is measured by taking the median Hounsfield Unit (HU) value of a nodule

^e^ According to the scoring system as presented in Table 3

### Eligibility criteria

**Fig. 2:**
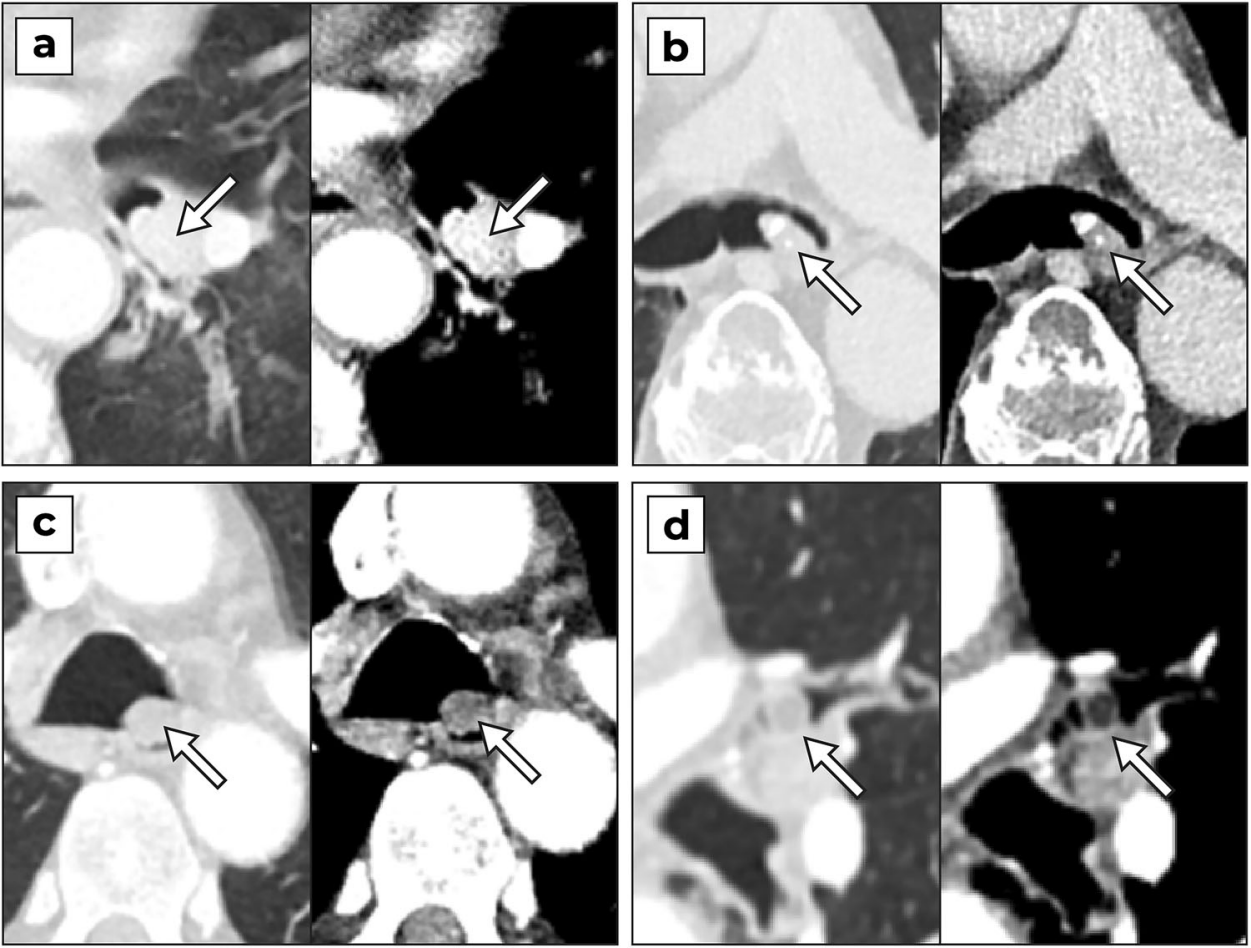
Examples of airway nodules in contrast-enhanced CT scans. For each panel, different window settings were applied for the lung (width: 1500 HU; level: −500 HU) and soft tissues (width: 350 HU; level: 0 HU). **a** A 60-year-old female patient with a carcinoid in the left lower bronchus (median attenuation value of 142 HU). **b** An 86-year-old male patient with a hamartoma with calcification in the left main bronchus (median attenuation value of 26 HU). **c** A 63-year-old male patient with nodular mucus plug in the distal trachea (median attenuation value of −18 HU). **d** A 57-year-old male patient with a lipoma in the left upper lobar bronchus (median attenuation value of −70 HU). HU, Hounsfield Units

All adult patients (≥ 18 years) with a chest or chest–abdomen CT scan (contrast-enhanced or non-enhanced) during the study period were eligible. Patients with airway nodules were defined as positive cases, and patients without any airway nodules were defined as negative cases. An airway nodule was defined as a focal opacity largely confined within the lumen of the airway (bronchus and trachea) [24]. Examples of airway nodules are provided in Fig. 2. Subsegmental mucus secretions were disregarded as nodules, because they are highly prevalent in a routine clinical setting with COPD and asthma patients [25, 26]. Patients with airway tumors were included unless the lesion was unmeasurable due to obstruction, atelectasis, and the lack of a contrast agent. Low-quality scans (i.e., severe breathing artifacts, missing slices, or thick slices > 3 mm) or scans with severely affected airways (i.e., extensive aspiration pneumonia or lung metastasis with bronchial infiltration or compression) were excluded.

### Reference standard

An experienced radiologist (E.T.S., 32 years of experience with chest CT) annotated all airway nodules in the dataset with in-house software (version 19.9.2 of CIRRUS Lung Screening, DIAG, Radboud University Medical Center). Nodules were segmented using a semi-automatic nodule segmentation algorithm [27] requiring manual localization and the segmentations were then corrected if necessary. Nodule locations were also determined with prior or follow-up CT scans, radiology reports, notes from bronchoscopy or surgery, and cancer information from the NCR. Nodule locations were categorized into the trachea, left or right main bronchus, or corresponding lung lobe for the smaller airways.

Primary cancers were verified through bronchoscopy with biopsy or cytologic testing. The malignancy status of other nodules was confirmed with bronchoscopy without further testing or follow-up CT scans if such evidence was unavailable. Bronchoscopy is the main diagnostic tool for airway tumors because it allows direct visualization of the tumor and tissue sampling [3]. However, the standard procedure is to conduct a follow-up CT scan first after an airway nodule has been detected, because nodular mucus secretions usually resolve over time and bronchoscopy is not required [24]. Table 2 and Supplementary Table 2 (Appendix 1) detail the basis of diagnosis and tumor morphology, respectively.

### Detection pipeline

The detection pipeline was based on the work of Hendrix et al [16] and was modified for the specific task of detecting airway nodules. The pipeline consists of three deep learning-based components for the following tasks: region of interest (ROI) detection (i.e., trachea, main bronchi, and lungs), nodule candidate detection, and false positive reduction. Given a CT scan as input, these tasks are automatically executed in consecutive order. The detection architecture was upgraded from YOLOv5 [28] to YOLOv8 [29] and the false positive reduction architecture from ResNet-50 [30] to ConvNeXt [31] for improved performance. Given the scarcity of training data, the components were pre-trained on the LUNA16 dataset [22] for transfer learning purposes. Appendix 3 provides technical details on the model architecture and training. The proposed system is publicly available for research purposes on the platform Grand-Challenge (https://grand-challenge.org/algorithms/airway-nodule-detection-for-routine-clinical-ct-sc/).

### Statistical analysis

The AI system was evaluated with a 10-fold cross-validation procedure to maximize the number of test samples. Each fold was divided into a training (90%) and test set (10%) using a stratified split based on lesion morphology (i.e., tumor vs non-tumorous) and location (i.e., trachea, primary bronchi, secondary bronchi, or more distal). The performance of the system was evaluated by measuring the sensitivity at predefined false positive rates on the free-response receiver operating characteristic (FROC) curve. For each operating point, the mean sensitivity and corresponding 95% confidence interval (CI) were calculated by using bootstrapping (1000 bootstraps using scan-level sampling with replacement). The optimal operating point on the FROC curve was selected where the sensitivity plateaus and the false positive rate starts to increase disproportionately. Given the low prevalence of airway nodules [24], false-positive rates were separately calculated for negative and positive patients. Separate analyses were conducted for nodule subsets with airway tumors and malignant tumors only. A predicted nodule was regarded as a true positive detection if its center was located within the convex segmentation of the ground truth nodule.

**Fig. 3:**
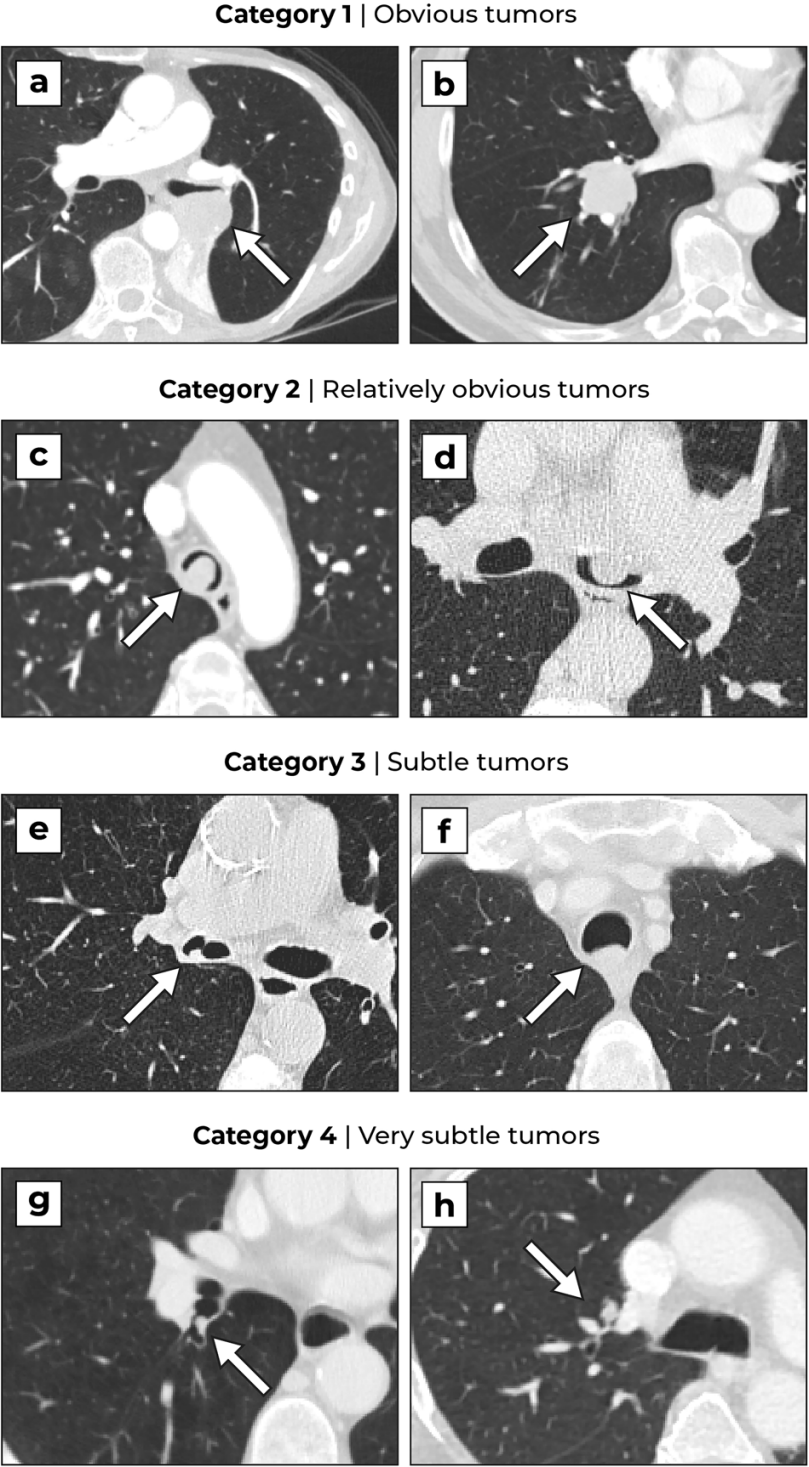
Examples of tracheobronchial tumors, ranked by subtleness. Obvious tumors: **a** A 73-year-old female patient with an adenosquamous carcinoma in the left lower lobe bronchus with evident post-obstruction atelectasis. **b** A 67-year-old male patient with a squamous cell carcinoma in the right lower lobe bronchus showing extensive extraluminal growth. Relatively obvious tumors: **c** A 67-year-old female patient with an adenoid cystic carcinoma in the trachea. **d** A 49-year-old male patient with an adenoid cystic carcinoma in the left main bronchus*.* Subtle tumors: **e** A 69-year-old male patient with a squamous cell carcinoma in the right main bronchus, not reported in the original examination. **f** A 62-year-old male patient with a squamous cell carcinoma in the trachea. Very subtle tumors: **g** A 65-year-old male patient with a squamous cell carcinoma at the ostium of the right lower lobe bronchus, not reported in the original examination. **h** A 60-year-old female patient with a hamartoma in the apical segment of the right upper lobe bronchus

Airway tumors were graded on subtleness to better assess the potential value of the AI system for radiological support. A scoring system was developed with an ordinal scale of four categories (obvious, relatively obvious, subtle, and very subtle) that were based on known characteristics of missed airway tumors [10–12]. An overview of the scoring system is shown in Table 3 and examples per category are shown in Fig. 3. Two experienced radiologists (E.T.S. and M.R., 32 years and 26 years of experience with chest CT, respectively) assessed the airway tumors independently. Then, a consensus reading was performed to resolve discrepancies. The sensitivity of the AI system was separately evaluated for each of the subtleness categories.

**Table 3:** Airway tumor scoring system

Category	Subtlety rating	Findings
1	Obvious^a^	Any evident tumor signs present: ▪ Post-obstruction atelectasis or consolidation ▪ Tumor grows into the lung parenchyma and its diameter is larger than surrounding bronchi or vessels
2	Relatively obvious^b^	▪ No evident tumor signs present from category 1 ▪ The nodule is located in the bronchus intermedius, main bronchus, or trachea ▪ The nodule occupies ≥ 75% of the local lumen
3	Subtle^c^	▪ No evident tumor signs present from category 1 ▪ The nodule is located in the bronchus intermedius, main bronchus, or trachea ▪ The nodule occupies < 75% of the local lumen
4	Very subtle^d^	▪ No evident tumor signs present from category 1 ▪ The nodule is located in a lobar bronchus or more distal

^a^ These tumor signs cover a large area and are therefore considered evident. Subsegmental tumors with substantial extraluminal growth can be detected by lung nodule CAD systems

^b^ Airway tumors are usually symptomatic when the lumen is narrowed by approximately 75% [31]

^c^ Airway tumors with limited luminal narrowing often remain clinically silent and therefore have a high chance of being overlooked as an incidental finding

^d^ Overlooked airway tumors are mostly located in the smaller airways [11, 12]. Maximum-intensity projection (MIP) techniques may obscure these nodules and cannot be used as a reading aid. In addition, mucus secretion is usually found in the smaller airways, thus lowering the radiologists’ vigilance

## Results

### Dataset characteristics

In the period of 2004–2020, 46,545 patients underwent a chest or chest–abdomen CT scan (see Fig. 1). From these patients, 6181 patients were excluded due to scans with a high slice thickness (> 3 mm). An airway nodule was found in 187 patients from which the malignancy status could be verified. Furthermore, 185 patients without airway nodules were randomly sampled. In both positive and negative groups, cases were excluded with extensive (post-obstruction) atelectasis or consolidations (*n*_pos_ = 10, *n*_neg_ = 12), poor scan quality (*n*_pos_ = 17, *n*_neg_ = 8), or severe pulmonary metastasis (*n*_neg_ = 5). The final selection contained 320 patients in total (median age of 62 years [IQR: 51–70], 138 women) with 160 negative patients (median age of 60 years [IQR: 48–69], 80 women) and 160 positive patients (median age of 64 years [IQR: 54–70], 58 women). The latter group consisted of 65 patients with primary airway cancer (67 lesions), 10 patients with airway metastasis (12 lesions), 13 patients with a benign airway tumor (13 lesions), and 78 patients with a non-tumorous airway nodule (94 lesions) (see Table 2).

### FROC curve analysis

**Fig. 4:**
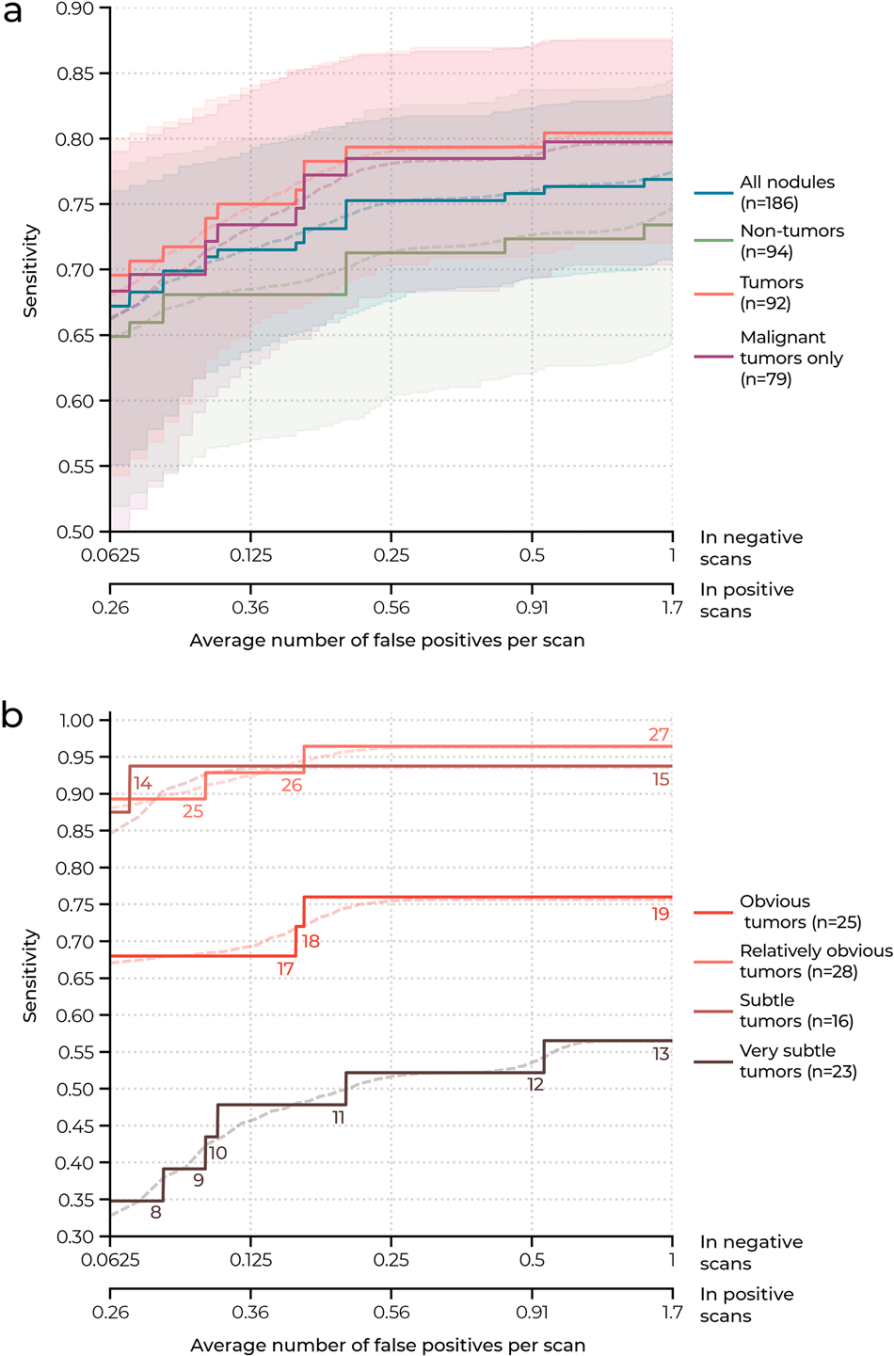
Free response receiver operating characteristic (FROC) curves of the AI system. The solid and dashed lines correspond with the observed sensitivities and mean sensitivities based on bootstrap samples, respectively. The shaded areas represent the 95% CIs. **a** FROC curves for all nodules and the subgroups tumors (benign and malignant) and malignant tumors only. **b** FROC curves for the tumor subcategories as presented in Table 3. The number of detected tumors is displayed near the FROC curves

Table 4 presents the sensitivity of the AI system at five predefined false positive rates in negative patients (0.0625, 0.125, 0.25, 0.5, and 1 false positive per scan on average) and matched false positive rates in positive patients. The corresponding FROC curves are shown in Fig. 4. Figure 4a shows the overall performance for all airway nodules, non-tumorous nodules, tumorous nodules, and malignant tumors only. Figure 4b shows the performance for tumor subgroups based on subtleness.

**Table 4:** Airway nodule detection results in negative (*n* = 160) and positive scans (*n* = 160)

Attribute	Operating points				
Threshold^a^	0.496	0.434	0.357	0.274	0.165
FP/s in negative scans	0.0625	0.125	0.250	0.500	1.00
FP/s in positive scans	0.256	0.360	0.560	0.910	1.72
Sensitivity—%					
All nodules (*n* = 186)	66.3 (55.1–76.0)	71.5 (63.8–79.1)	75.1 (67.6–81.6)	76.1(69.3–82.4)	77.4 (70.8–83.4)
Non-tumors (*n* = 94)	64.8 (51.4–77.5)	68.5 (56.9–80.0)	71.4 (60.3–82.3)	72.7 (62.1–83.7)	74.7 (64.7–84.4)
Tumors (*n* = 92)	68.0 (54.3–80.0)	74.7 (65.4–84.0)	79.0 (70.4–86.6)	79.6 (71.2–87.0)	80.3 (72.0–87.6)
Malignant tumors only (*n* = 79)	66.2 (50.0–79.0)	73.1 (62.7–83.5)	78.1 (68.2–86.4)	78.8 (69.4–87.2)	79.6 (70.4–87.5)
Sensitivity per tumor category—% ^b^					
Obvious (*n* = 25)	67.0 (48.0–85.0)	69.3 (50.0–88.1)	75.4 (57.1–92.0)	75.7 (57.7–92.0)	75.7 (57.7–92.0)
Relatively obvious (*n* = 28)	88.1 (74.1–100)	92.4 (81.2–100)	96.2 (87.5–100)	96.3 (88.0–100)	96.3 (88.0–100)
Subtle (*n* = 16)	84.6 (53.3–100)	93.4 (77.5–100)	93.6 (78.9–100)	93.6 (78.9–100)	93.6 (78.9–100)
Very subtle (*n* = 23)	32.8 (10.5–57.1)	45.6 (23.4–68.2)	51.7 (31.1–72.2)	53.6 (31.8–74.9)	56.5 (35.3–76.9)

95% CI are in parentheses

*FP/s* average number of false positives per scan

^a^ Estimated threshold of the model output (i.e., nodule likelihood score)

^b^ According to the scoring system as presented in Table 3

The sensitivity of the AI system was 75.1% (95% CI: 67.6–81.6%) for detecting all airway nodules with 0.25 and 0.56 false positives per scan (FP/s) on average in negative and positive patients, respectively. The sensitivity for detecting non-tumorous nodules was 71.4% (95% CI: 60.3–82.3%) with the same false positive rates. The sensitivities for detecting tumorous nodules and malignant tumors only (primary cancers and metastases) were 79.0% (95% CI: 70.4–86.6%) and 78.1% (95% CI: 68.2–86.4%), respectively. The sensitivity per tumor subtlety subgroup is included in Table 4.

### Detection examples

**Fig. 5:**
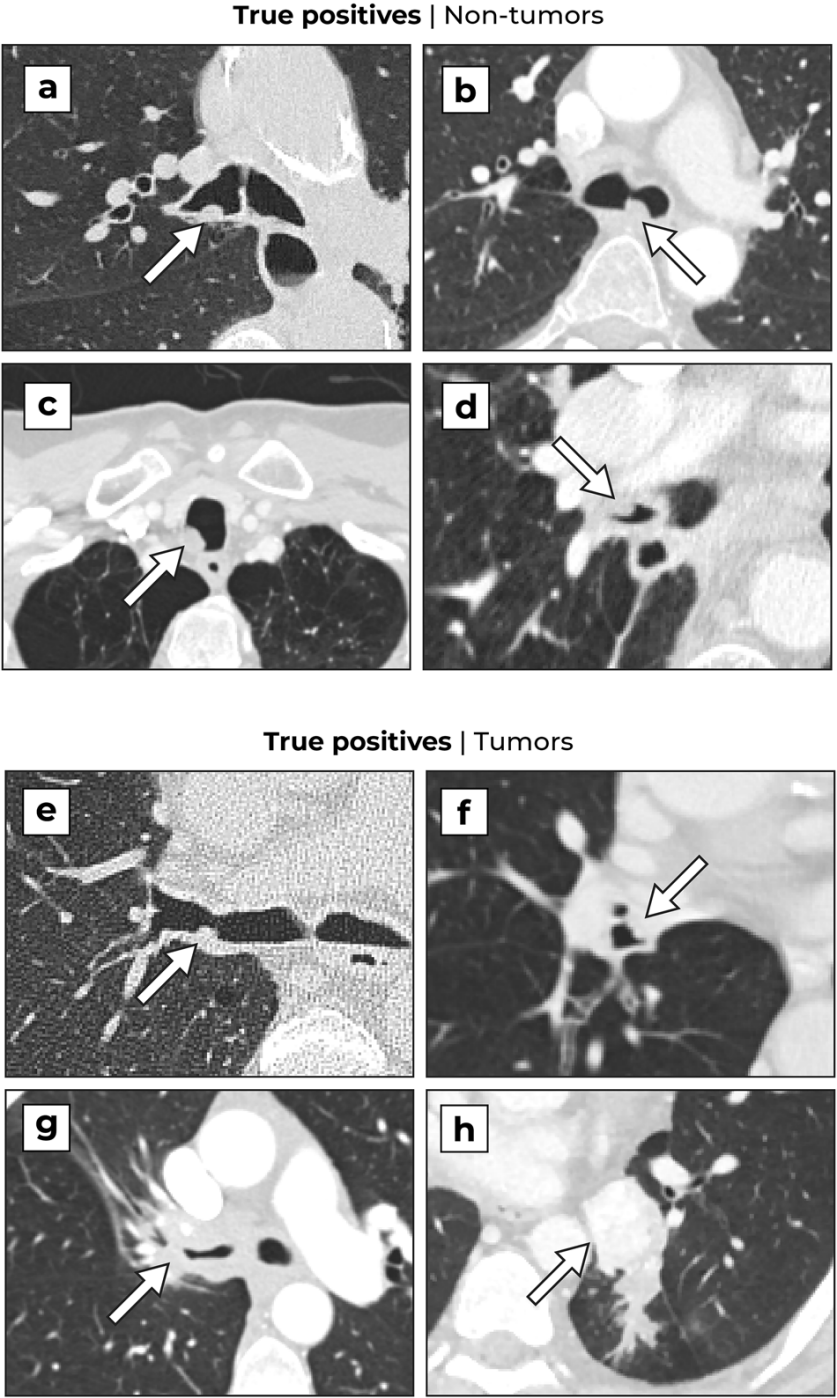
Examples of true positives of the AI system. Non-tumors: **a** A 72-year-old female patient with a mucus plug in the right main bronchus. **b** A 51-year-old male patient with a mucus plug in the trachea bifurcation, left the main bronchus. **c** A 64-year-old male patient with a mucus plug in the proximal trachea. **d** A 68-year-old male patient with a mucus plug in the right lower lobe bronchus. Tumors: **e** A 68-year-old male patient with a squamous cell carcinoma at the ostium of the right upper lobe bronchus. **f** A 63-year-old male patient with a squamous cell carcinoma at the ostium of the right lower lobe bronchus, not reported in the original examination. **g** A 42-year-old female patient with an adenoid cystic carcinoma of the trachea and right main bronchus. **h** A 50-year-old male patient with a carcinoid in the left lower bronchus

**Fig. 6:**
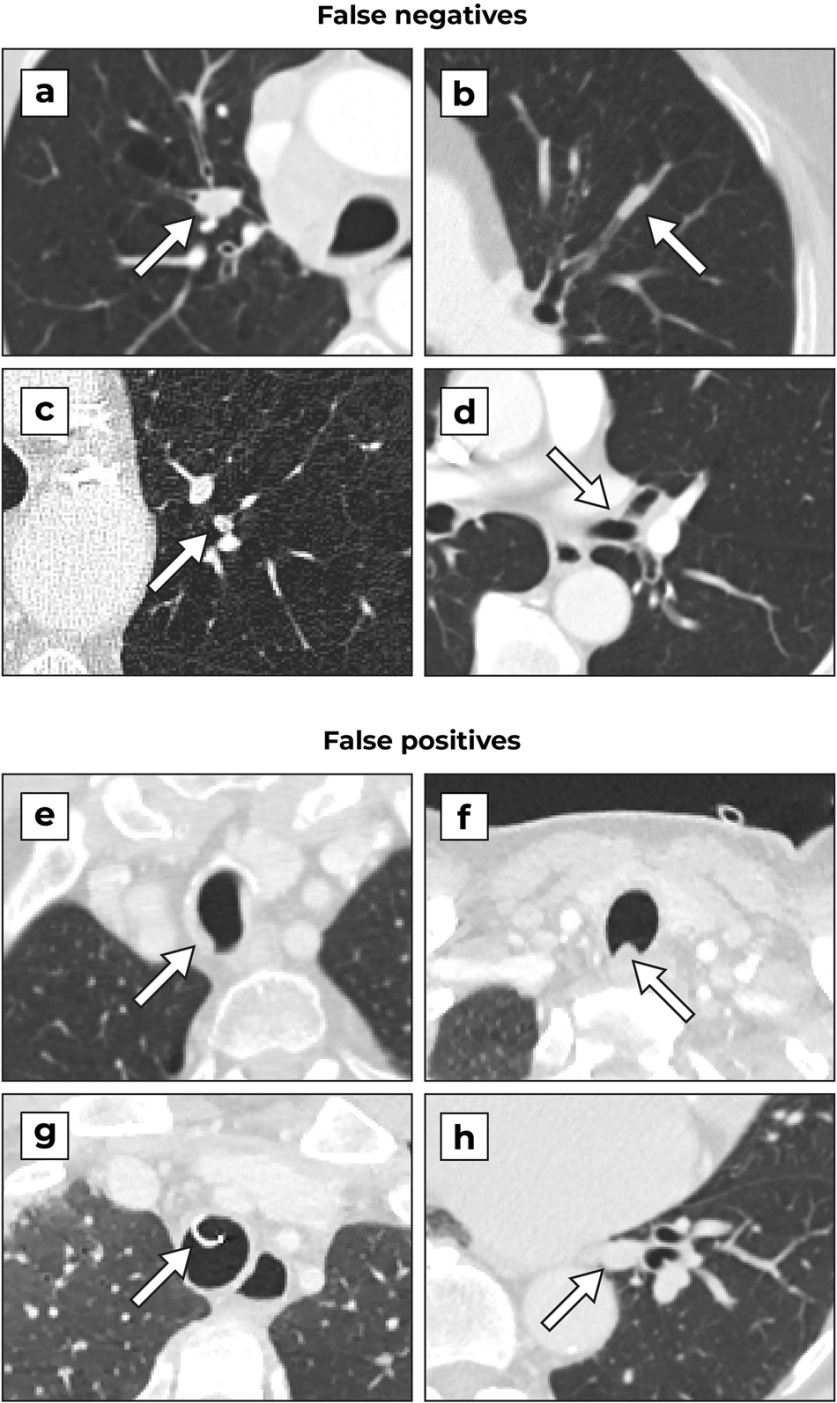
Examples of false negatives and positives of the AI system. False negatives: **a** A 58-year-old female patient with a metastasis of a Hurthle cell carcinoma in the apical segment of the right upper lobe bronchus. **b** An 85-year-old male patient with a non-small cell carcinoma in a subsegmental bronchus in the left upper lobe. **c** A 68-year-old male patient with a squamous cell carcinoma in a subsegmental bronchus in the left upper lobe, not reported in the original examination. **d** A 67-year-old male patient with a duct carcinoma at the ostium of the left upper lobe bronchus. False positives: **e** A 78-year-old male patient with non-nodular mucus secretion in the trachea. **f** A 28-year-old male patient showing anterior bowing of the posterior trachea membrane. **g** A 23-year-old male patient with a trachea tube. **h** A 75-year-old male patient with detection on the left inferior pulmonary vessel

Examples of true positive detections on non-tumors and tumors at an operating point of 0.25 FP/s in negative patients are shown in Fig. 5. Common failure modes of the DL-CAD system at the same operating point are illustrated in Fig. 6. False negatives were typically cancers in the lobar bronchi or more distal (panels a–d), especially when located in a subsegmental bronchus. Most false positive detections were observed on non-nodular mucus secretions (panel e). Other false positives were detections on the anterior bowing of the posterior tracheal membrane (panel f), trachea tubes (panel g), or pulmonary vessels in the hilar region (panel h). On some occasions, the AI systems also detected relevant abnormalities other than airway nodules, namely intrapulmonary nodules, or esophageal lesions (when close to the airways or lungs).

## Discussion

Airway tumors are rare and are recognized as a common blind spot for radiologists [10–13]. Given the recent advancements in AI, our aim was to develop a DL-CAD system for the detection of airway nodules and to assess its technical performance in a routine clinical setting. We demonstrate that a DL-CAD system can detect 75% of all airway nodules with an average of 0.25 FP/s in negative patients, even with limited training data available. For the subset of benign and malignant tumors, the sensitivity was higher and reached 79% for the same false positive rate. A subgroup analysis showed that the system can also detect most of the subtle tumors that are hard to detect by radiologists.

To the best of our knowledge, this is the first study that investigates the performance of a DL-CAD system for airway nodules. The FROC analysis (Fig. 4a) shows that the detection performance is higher for tumorous lesions compared to non-tumorous lesions (average difference of 6 percentage points across all operating points). This can be explained by the larger diameter of the tumors (a median of 13.4 mm compared to 7.1 mm). Nodular mucus secretions may also be more difficult to detect due to their more obtuse angle with the bronchus wall and lower contrast compared to tumors (except for lipoma), as illustrated in Fig. 2. Although the main objective is to detect tumors, it is still important to also detect nodular mucus secretions as they can be difficult to distinguish from tumors (see Fig. 5a–d). A radiologist can assess the nodule based on the presence of internal air [32] and decide whether further clinical evaluation is needed with repeated CT imaging after vigorous coughing or bronchoscopy.

The FROC analysis in Fig. 4b shows that the DL-CAD system has a lower detection performance for very subtle tumors compared to more obvious tumors. It is possible that the DL-CAD system rejects these lesions as subsegmental mucus secretion or intrapulmonary nodules, which were not annotated in the dataset (see “Eligibility criteria” in the “Materials and methods” section). The (sub)segmental airways therefore remain an area of attention for both radiologists and DL-CAD systems. Nonetheless, the DL-CAD system can already detect and correctly localize the majority of (very) subtle tumors and therefore has potential clinical value.

Paradoxically, the system has a lower sensitivity for obvious tumors compared to relatively obvious and subtle tumors. The most salient tumors are often accompanied by post-obstruction atelectasis and have a less nodular appearance overall (see Fig. 3a, b), which could explain why these tumors are harder to detect. The system still has a high sensitivity for tumors which were, at least in retrospect, relatively obvious. These tumors can be overlooked on CT scans in cases of inattentional blindness [11, 33] and a DL-CAD system may be useful in such situations.

A recommendation for future research is to investigate the optimal operating point for airway nodule DL-CAD systems. As there are no prior studies on this topic, we chose an operating point that balances sensitivity and specificity, but this ultimately depends on the cost-benefit tradeoff. For instance, the DL-CAD system has a high sensitivity for tumors in the large airways, and a radiologist may accept a false positive CAD mark every few scans to reduce the effort of screening this area. However, if these false positives result in a significant increase in unnecessary follow-up examinations, the associated costs may outweigh the benefits of early detection and the false positive rate may need to be reduced.

This study has several limitations. First, the proposed DL-CAD system was not evaluated on an external validation set, as we did not have an external dataset with airway tumors available. Nonetheless, the algorithm has been trained and evaluated on CT scans with a wide variety of scanning protocols (see Table 1 and Supplementary Table 1 in Appendix 1) and the results are therefore expected to generalize to other institutions. The fact that our algorithm is publicly available provides the opportunity for further external validation by other researchers in the future. Second, the performance of the DL-CAD system was likely limited by the small number of available training samples. Kim et al [24] and DeSimone et al [21] found a prevalence of airway nodules of 0.6% (313/53,036) and 2% (178/9068) in a low-dose CT screening cohort, respectively. We found a similar prevalence in our routine clinical cohort, which suggests that the small number of samples is inevitable unless data is collected from many institutions. Third, we did not conduct an observer study to compare the performance of the DL-CAD system with that of radiologists. An unbiased, retrospective evaluation can be difficult, because airway nodules are a rare appearance, and they will inevitably increase the radiologists’ vigilance if they are not presented with their natural incidence. We used a rating system based on known overlooked tumors to mitigate this issue and assess the potential value of the DL-CAD system.

In conclusion, we found that a DL-CAD system can detect most benign and malignant airway nodules in routine clinical chest CT scans with an acceptable false positive rate. Despite the rarity of these nodules, our findings demonstrate the technical feasibility of developing a DL-CAD system that could be useful for radiologists in real-world clinical settings. Future research should aim to evaluate the performance of the DL-CAD system in a larger clinical cohort from multiple institutions.

## Supplementary information

ELECTRONIC SUPPLEMENTARY MATERIAL

## Abbreviations

AI: Artificial intelligence
COPD: Chronic obstructive pulmonary disease
DL-CAD: Deep learning-based computer-aided detection
FP/s: False positives per scan
FROC: Free-response receiver operating characteristic
NCR: Netherlands Cancer Registry
NLP: Natural Language Processing
