# Supplementary material for: Artificial intelligence for the detection of airway nodules in chest CT scans
Source: Eur Radiol. 2025 Mar 5;35(9):5615–25. doi: 10.1007/s00330-025-11468-6 (PMC12350537; doi:10.1007/s00330-025-11468-6)
Supplement: Supplementary file 1 — ELECTRONIC SUPPLEMENTARY MATERIAL [file 330_2025_11468_MOESM1_ESM.pdf]

Artificial Intelligence for the Detection of Airway Nodules in Chest CT Scans

Electronic Supplementary Material

Eur Radiol (2025) Hendrix W, Hendrix N, Scholten ET, et al.

Appendix 1 – Additional dataset characteristics

Supplementary Table 1. Additional imaging parameters.

Characteristic	All	With airway nodules	Without airway nodules
No. of CT scans	210	160	50
Peak potential energy in kVp – median (IQR)	120 (100-120)	120 (100-120)	120 (100-120)
Tube current in mAs – median (IQR)	315 (165-451)	307 (165-451)	319 (157-463)
CT scans per scanner – no. (%)			
GE Medical Systems Discovery CT750 HD	5 (2.4)	4 (2.5)	
Medical Systems Optima CT660	4 (1.9)	4 (2.5)	
Philips Brilliance 40	2 (1.0)	2 (1.3)	
Philips Brilliance 64	7 (3.3)	5 (3.1)	2 (4.0)
Philips MX8000 IDT 16	2 (1.0)	2 (1.3)	
Philips iCT 256	5 (2.4)	3 (1.9)	2 (4.0)
Siemens Biograph 40	5 (2.4)	4 (2.5)	1 (2.0)
Siemens Emotion Duo	11 (5.2)	7 (4.4)	4 (8.0)
Siemens Somatom Definition AS	12 (5.7)	8 (5.0)	4 (8.0)
Siemens Somatom Definition AS+	3 (1.4)	3 (1.9)	
Siemens Somatom Definition Flash	7 (3.3)	7 (4.4)	
Siemens Sensation 16	61 (29.0)	42 (26.3)	19 (38.0)
Siemens Sensation 64	18 (8.6)	14 (8.8)	4 (8.0)
Canon Aquilion CXL	17 (8.1)	15 (9.4)	2 (4.0)
Canon Aquilion One	35 (16.7)	25 (15.6)	10 (20.0)
Canon Aquilion Precision	7 (3.3)	7 (4.4)	
Other	9 (4.3)	8 (5.0)	2 (4.0)

Note. The Other category contains less than 5% of all scans.

Supplementary Table 2. Tumor morphology.

Characteristic	Primary airway cancer	Airway metastasis	Benign airway tumor
No. of patients	65	10	13
Morphology – no. (%) of patients			
Adenocarcinoma, NOS	1 (1.5)	2 (20.0)	
Adenoid cystic carcinoma	12 (18.5)		
Adenosquamous carcinoma	1 (1.5)		
Atypical carcinoid tumor	2 (3.1)		
Clear cell adenocarcinoma, NOS		1 (10.0)	
Duct carcinoma, NOS	1 (1.5)		
Glomangioma			1 (7.7)
Hamartoma			4 (30.8)
Hurthle cell carcinoma		1 (10.0)	
Inflammatory myofibroblastic tumor			1 (7.7)
Leiomyoma			1 (7.7)
Lipoma			6 (46.2)
Mucinous adenoma	1 (1.5)		
Mucoepidermoid carcinoma	2 (3.1)		
Myosarcoma		1 (10.0)	
Neoplasm, NOS	2 (3.1)		
Neuroendocrine tumor, grade I	20 (30.8)		
Non-small cell carcinoma	1 (1.5)		
Papillary adenocarcinoma, NOS	1 (1.5)		
Renal cell carcinoma		2 (10.0)	
Small-cell lung carcinoma		1 (10.0)	
Squamous cell carcinoma, keratinizing	5 (7.7)		
Squamous cell carcinoma, large cell, nonkeratinizing	3 (4.6)		
Squamous cell carcinoma, NOS	14 (21.5)	1 (10.0)	
Superficial spreading melanoma		1 (10.0)	

Note. One patient had two primary airway cancers and was counted twice. *CIS* = Carcinoma in situ; *NOS* = Not otherwise specified, see ICD-O [1].

Appendix 2 – Natural language processing analysis

Background

Natural language processing (NLP) was used to pre-select radiology reports with reported airway nodules. Unlike pulmonary nodules, we observed that airway nodules are less consistently described as “nodules” or “endobronchial” entities. It is possible that non-specialized radiologists are less familiar with these nodules due to their rarity [2]. Given the ambiguity in the description of incidental airway nodules, we chose to pre-select patients using deep learning-based NLP methods instead of conventional keyword searches. The initial screening was done by author W.H. in consultation with radiologist E.T.S. with experience with assessing endobronchial nodules [3].

Semantic search

A first set of relevant radiology reports was obtained by a semantic NLP search engine that returns relevant findings related to a set of input phrases (e.g., “endobronchial nodule”). This method returns similar findings even when the keywords do not exactly match. Our dataset contained 135,918 radiology reports (46,545 patients) from chest CT examinations from the period 2004-2020. The processing steps were as follows:

1. **Extract impression sentences:** Extract the impression section from each radiology report with regular expressions and split the text into sentences. This procedure significantly reduces the length of the documents while only the most relevant radiological findings are kept.
2. **Create sentence embeddings:** Tokenize the sentences and convert the tokens into word embeddings (128 dimensions) with the Word2Vec algorithm [4] using the continuous bag-of-words (CBOW) architecture. Sentence embeddings are created by averaging word embeddings with a TF-IDF (Term Frequency – Inverse Document Frequency) weighting scheme.

3. **Pre-select relevant findings**: Create a list of input queries with relevant keywords (e.g., “endobronchial nodule”, “intraluminal lesion”, “trachea tumor”, etc.) and match all unique sentences with a cosine similarity score of at least 0.4. This step returned approximately 10,000 hits.
4. **Manually check findings:** Sort the sentences on semantic similarity (descending) and manually check the sentences until they are no longer relevant. In our case, we checked the 500 most relevant findings and subsequently verified them by reading the complete radiology reports.

Text classification

A text classification model was developed for a more exhaustive search in our text corpus. A BERT (transformer) model was trained on the initial results from the semantic search procedure (see previous section). We chose a pre-trained Dutch BERT model [5] that has been implemented in the spaCy library (version 3.5.3, 2023) for Python. Only the history and impression sections of the radiology reports were used to reduce the document length and to focus on the most relevant findings. We defined a multi-label classification task where reports were labelled with “solid airway nodules”, “mucus secretion”, or none of these labels. Ambiguously reported nodules were labeled as both solid and mucus secretion.

The model was iteratively updated by adding new search results to the training dataset. Predictions were sorted on classification probability (descending) and the corresponding radiology reports were manually checked until they were no longer relevant. Patients with known primary lung cancer or metastasis were prioritized in this process and completely checked. For each iteration, we used an 80/20 split for training and validation and oversampled the minority class. The default configuration settings were used as provided by the spaCy library (from the Dutch “nl_core_news_lg” training pipeline).

The final iteration of the text classification model was trained with 2,520 reports (459 with airway mucus, 714 with airway solid nodules, the other reports are negative). A test set was created with 140 reports in total, including 50 reports with solid airway nodules and 50

reports with mucus secretions. Reports in this dataset were randomly sampled from the initial search results (see previous section) to prevent sampling bias and they were stratified by location (i.e., trachea, main bronchi, other bronchi). There was no patient overlap with the training dataset. On the test dataset, the NLP model obtained AUROC (Area Under the Receiver Operating Characteristic Curve) score of 0.98 for mucus secretion and 0.91 for solid nodules. For a single operating point (maximum F1-score), the precision and recall were 96% and 98% for mucus secretion and respectively 82% and 84% for solid nodules. For any airway nodule (binary), the AUROC score was 0.89 and the precision and recall scores were respectively 94% and 89%.

Final data selection

For the final data selection, all radiology reports from a patient were examined by author E.T.S. with the corresponding CT scans and additional information (e.g., biopsy and surgical information) as described in the main text (see section Reference standard). The NLP analysis was not used for selecting patients with known cancer in the trachea, carina, or main bronchi (including bronchus intermedius). These patients could be accurately pre-selected with ICD-O topology codes C339 and C340 as provided by the Netherlands Cancer Registry (NCR). Code C340 also encompasses rare hilar lung cancers [6], which were manually removed from our dataset.

Appendix 3 – Model architecture description and training procedures

The airway nodule detection pipeline in this study was adapted from the work of Hendrix et al. on pulmonary nodule detection in routine clinical CT scans [7]. The pipeline consists of three components for the following tasks: (a) region-of-interest (ROI) detection, (b) nodule candidate detection, and (c) false positive reduction. This configuration is commonly used for pulmonary nodule detection [8]. Changes in the model architectures and training procedures are outlined in the next sections.

Changes to model architecture

Region-of-interest detection

The ROI component detects the trachea, bronchi, and lungs in order to reduce the CT volume for the subsequent components. The architecture of this component was upgraded from YOLOv5 [9] to YOLOv8 (release of 12 November 2023) [10] for increased performance. YOLOv8 is an anchorless version of YOLOv5 and obtains a higher detection score on the COCO benchmark dataset [11], an improvement of 6% to 33% depending on the model size. A small model was chosen for all experiments ("YOLOv8s"). No other changes to the architecture or configuration were made.

Nodule candidate detection

The aim of the nodule candidate detection component is to detect all possible nodule locations in the scan regardless of the false positive rate. The architecture of this component was also upgraded from YOLOv5 to YOLOv8. An instance segmentation variant was chosen for more accurate localization of the nodule center, especially in complex shapes. A medium model was chosen for all experiments ("YOLOv8m-seg"). The image resolution was reduced from 1024 x 1024 pixels to 512 x 512 pixels to reduce memory usage. Feature maps from the P2 layer were propagated to the detection head to compensate for the change in

resolution, which is important for the detection of very small objects [12]. No other changes to the architecture were made.

False positive reduction

The false positive reduction component reduces the false positive rate while retaining a high sensitivity. The model was adapted from Venkadesh et al. [13] and it processes nine different 2D views from a 3D patch around the nodule candidate. We upgraded the architecture from ResNet-50 [14] to ConvNeXt [15] for improved performance. The architecture of ConvNeXt is based on a standard ResNet, but it is "modernized" towards the design of a Vision Transformer (ViT) [16]. We chose the tiny model variant ("ConvNeXt-T") that has approximately the same number of parameters as a ResNet-50 model. No other changes to the architecture were made.

Model pre-training

The whole detection pipeline was pre-trained on CT scans from the publicly available LUNA16 dataset [17]. This dataset contains a subset of 888 CT scans from the LIDC-IDRI archive [18]. Re-encoded lung nodule segmentations from Federov et al. [19] were used for generating training samples. Labels from at least one radiologist (out of four) were used for training, and labels from at least three radiologists were used for validation. ROI bounding box annotations were made by author W.H. according to the same protocol as our previous study [7]. The dataset was split into a training (80%) and validation set (20%) for hyperparameter tuning and model checkpoint selection. The training procedure of all model components was halted when the detection or classification performance did no longer improve on the validation set.

Region-of-interest detection

During training, the model checkpoint with the best fitness score was selected. The default fitness score is a weighted combination of mean average precision (mAP) scores at different

intersection over union (IoU) thresholds. We used a batch size of 16 and applied the default YOLOv8 training settings and data augmentations, except for mosaic, horizontal flips, and color augmentations. During inference, the confidence threshold was set to 0.4 and IoU threshold to 0.6. Bounding boxes were converted to segmentation masks and resampled to an isotropic voxel spacing of 1 x 1 x 1 mm. Consequently, a dilation operation was applied with large kernel size of 11 to close any gaps. The largest connected component (class agnostic) was selected as the region-of-interest, so that detection errors outside the chest region were removed.

Nodule candidate detection

During training, the model checkpoint with the best fitness score was selected as described in the previous section. We used a batch size of 8 and applied the default YOLOv8 training settings and data augmentations, except for mosaic and color augmentations. Rotation augmentations (from -20 to +20 degrees) were added to the training procedure to increase the robustness of the model. The mask ratio was set to 1. During inference, slice-by-slice predictions were merged using a connected component analysis instead of DBSCAN [20] due to the use of segmentations.

False positive reduction

During training, the model checkpoint with the best area under the precision recall curve (PR-AUC) was selected. Horizontal flips (50% probability) were added to training procedure to increase model performance. We set the batch size to 64 and used an SGD optimizer with Nesterov momentum (learning rate = 1e-3, momentum of 0.9). All nodule candidates with a minimum probability threshold of 1e-3 were selected. Nodule candidates within the 3D bounding box of the ROI were kept during training, but during inference, candidates were pruned by removing those outside the ROI masks.

Model training

As described in the main text (section Detection pipeline), the whole detection pipeline was trained and evaluated using a 10-fold cross validation procedure. Each fold was split into a training (90%) and test set (10%) in a stratified fashion based on lesion morphology (i.e., tumor vs non-tumorous) and location (i.e., trachea, primary bronchi, secondary bronchi or more distal). Given the large imbalance in nodule locations (see Table 2 in the main text), a stratified random sampler was used during training of the nodule candidate detection and false positive reduction components. Each model was trained until convergence. No other changes were made to the training procedure as described in the pre-training section.

References

1. World Health Organization (2013) International classification of diseases for oncology (ICD-O) 3rd ed. Fritz A, Percy C, Jack A, et al., editors. Switzerland: World Health Organization
2. Kim H-J, Kim DK, Kim YW, et al. (2016) Outcome of incidentally detected airway nodules. Eur Respir J DOI: 10.1183/13993003.01992-2015
3. Scholten ET, Horeweg N, de Koning HJ, et al. (2015) Computed tomographic characteristics of interval and post screen carcinomas in lung cancer screening. Eur Radiol DOI: 10.1007/s00330-014-3394-4
4. Mikolov T, Chen K, Corrado G, Dean J (2013) Efficient Estimation of Word Representations in Vector Space. arXiv DOI: 10.48550/arXiv.1301.3781
5. de Vries W, van Cranenburgh A, Bisazza A, Caselli T, van Noord G, Nissim M (2019) BERTje: A Dutch BERT Model. arXiv DOI: 10.48550/arXiv.1912.09582
6. Watanabe Y, Shimizu J, Oda M, et al. (1991) Early hilar lung cancer: Its clinical aspect. J Surg Oncol DOI: 10.1002/jso.2930480202
7. Hendrix W, Hendrix N, Scholten ET, et al. (2023) Deep learning for the detection of benign and malignant pulmonary nodules in non-screening chest CT scans. Commun Med DOI: 10.1038/s43856-023-00388-5
8. Gu Y, Chi J, Liu J, et al. (2021) A survey of computer-aided diagnosis of lung nodules from CT scans using deep learning. Comput Biol Med DOI: 10.1016/j.compbiomed.2021.104806
9. Jocher G, Stoken A, Borovec J, et al. (2021) ultralytics/yolov5: v5.0 - YOLOv5-P6 1280 models, AWS, Supervise.ly and YouTube integrations. Zenodo DOI: 10.5281/zenodo.4679653
10. Jocher G, Chaurasia A, Qiu J (2023) Ultralytics YOLOv8. Ultralytics https://github.com/ultralytics/ultralytics. Accessed November 23, 2023
11. Lin T-Y, Maire M, Belongie S, et al. (2015) Microsoft COCO: Common Objects in Context. arXiv DOI: 10.48550/arXiv.1405.0312

12. Benjumea A, Teeti I, Cuzzolin F, Bradley A (2023) YOLO-Z: Improving small object detection in YOLOv5 for autonomous vehicles. arXiv DOI: 10.48550/arXiv.2112.11798
13. Venkadesh KV, Setio AAA, Schreuder A, et al. (2021) Deep Learning for Malignancy Risk Estimation of Pulmonary Nodules Detected at Low-Dose Screening CT. Radiology DOI: 10.1148/radiol.2021204433
14. He K, Zhang X, Ren S, Sun J (2016) Deep Residual Learning for Image Recognition. 2016 IEEE Conference on Computer Vision and Pattern Recognition (CVPR) DOI: 10.1109/CVPR.2016.90
15. Liu Z, Mao H, Wu C-Y, Feichtenhofer C, Darrell T, Xie S (2022) A ConvNet for the 2020s. 2022 IEEE/CVF Conference on Computer Vision and Pattern Recognition (CVPR) DOI: 10.1109/CVPR52688.2022.01167
16. Dosovitskiy A, Beyer L, Kolesnikov A, et al. (2021) An Image is Worth 16x16 Words: Transformers for Image Recognition at Scale. arXiv DOI: 10.48550/arXiv.2010.11929
17. Setio AAA, Traverso A, de Bel T, et al. (2016) Validation, comparison, and combination of algorithms for automatic detection of pulmonary nodules in computed tomography images: the LUNA16 challenge. Med Image Anal DOI: 10.1016/j.media.2017.06.015
18. Armato SG, McLennan G, Bidaut L, et al. (2011) The Lung Image Database Consortium (LIDC) and Image Database Resource Initiative (IDRI): A completed reference database of lung nodules on CT scans. Med Phys DOI: 10.1118/1.3528204
19. Fedorov A, Hancock M, Clunie D, et al. (2020) DICOM re-encoding of volumetrically annotated Lung Imaging Database Consortium (LIDC) nodules. Med Phys DOI: 10.1002/mp.14445
20. Ester M, Kriegel H-P, Sander J, Xu X (1996) A density-based algorithm for discovering clusters in large spatial databases with noise. Proceedings of the Second International Conference on Knowledge Discovery and Data Mining. AAAI Press; p. 226–231.
